# Effects of a *Saccharomyces cerevisiae* Fermentation Product on Diet Palatability and Feline Intestinal Health, Immunity, and Microbiome

**DOI:** 10.3390/ani15172551

**Published:** 2025-08-30

**Authors:** Patricia Eri Ishii, Fabio Alves Teixeira, Ching-Yen Lin, Syed Ali Naqvi, Maria I. Sardi, Sharon A. Norton, Jessica K. Jarett, Ehsan Khafipour, Nolan Frantz, Anirikh Chakrabarti, Jan S. Suchodolski

**Affiliations:** 1 Gastrointestinal Laboratory, Department of Small Animal Clinical Sciences, Texas A&M University, College Station, TX 77843, USA; pishii@stonewell.com.br (P.E.I.); jsuchodolski@cvm.tamu.edu (J.S.S.); 2 Stonewell Gastrointestinal Laboratory, São Paulo 04041-004, Brazil; 3 School of Veterinary Medicine and Animal Science, University of São Paulo, São Paulo 05508-270, Brazil; 4 Blue Buffalo Company, Ltd., Wilton, CT 06897, USAnfrantz@bluebuff.com (N.F.); 5 Cargill Incorporated, Wayzata, MN 55391, USA; naqvi.ali.92@gmail.com (S.A.N.); maria_sardi@cargill.com (M.I.S.); nortsh1@gmail.com (S.A.N.); jess_jarett@cargill.com (J.K.J.); ehsan.khafipour@gmail.com (E.K.); anirikh_chakrabarti@cargill.com (A.C.)

**Keywords:** cat, nutrition, immuno-nutrition, postbiotic, gut microbiome

## Abstract

This study evaluated the effects of adding a *Saccharomyces cerevisiae* fermentation product (SCFP) to adult cat diets on palatability, intestinal health, nutrient digestibility, immune parameters, and the fecal microbiome over 42 days. Sixty-three healthy Domestic Short-hair cats were randomized to three diets: a control diet (CD) without SCFP, or the same diet containing 1.0% or 2.0% SCFP, targeting daily intakes of 150 or 300 mg/kg body weight, respectively. Body weight and blood parameters remained within reference ranges across all groups. Stool quality was largely not affected, although compared with controls, SCFP-supplemented cats had slightly firmer stools at day 21, and increased fecal pH. Shotgun metagenomic sequencing revealed that microbiome diversity remained steady in SCFP-fed cats, whereas diversity in the control group declined over time. Fecal immunoglobulin A concentrations were lower in cats supplemented with SCFP at 150 mg/kg BW by the end of the study, and positive shifts in the circulatory leukocyte profile were observed at both inclusion levels. Apparent total tract macronutrient digestibility did not differ among groups. Palatability tests showed diets with SCFP were generally preferred, indicating a potential benefit for commercial feline feeds, particularly at the 150 mg/kg BW level, which was preferred over 300 mg/kg BW. Overall, these findings suggest that SCFP can act as a functional ingredient in feline nutrition to maintain microbial diversity and enhance diet acceptance without compromising digestibility.

## Simple Summary

Cats often experience digestive and immune challenges that can affect their well-being. This study investigated whether adding *Saccharomyces cerevisiae* fermentation product (SCFP) to cat food could improve health and food preference. Over six weeks, three groups of 21 healthy cats were fed a standard diet or the same diet with two different amounts of SCFP. The cats accepted the supplemented foods, and those receiving the SCFP tended to prefer it, especially at the intermediate level tested. Their stool quality remained good, with slight improvements at one point, and all blood values stayed within the normal range. Cats eating the SCFP-supplemented diets maintained a more stable community of beneficial gut microbes, while cats on the standard diet showed a decline in microbial diversity over time. Cats on the supplemented foods had more immune cells that help fight infections over time, and fewer of those that respond to short-term stress or allergies. The inclusion of SCFP did not affect nutrient digestibility. Overall, adding this supplement to cat diets appears to help preserve a healthy gut environment and may improve food acceptance, offering a promising option for supporting the health of companion cats. Further studies are needed to clarify some findings.

## 1. Introduction

In recent years, there has been a growing interest in the potential health benefits of postbiotics derived from *Saccharomyces cerevisiae* fermentation products (SCFP). This is achieved by inoculation of live yeast cells into a specific culture medium, followed by controlled fermentation and drying of fermentation metabolites [1]. This dry product includes residual yeast cells, yeast cell wall fragments, fermentation metabolites as a result of anabolic or catabolic activities of yeast, and media used during fermentation, and may serve as a functional ingredient due to its benefits to animals [2]. Thus, SCFP can be considered postbiotics, defined by the International Scientific Association for Probiotics and Prebiotics as the “preparation of inanimate microorganisms and/or their components that confers a health benefit on the host” [3].

Postbiotics, in general, have been shown to positively impact animal health and specifically gut health across different animal species [4,5,6,7,8]. While researchers continue to unravel the mechanism of action of postbiotics, there is growing consensus on several areas of positive impact, including regulation of immunity, strengthening the gut barrier, inhibition of pathogens, and promoting a healthy gut microbiome [7]. Postbiotics from SCFP are used in companion- and food-producing animals for their beneficial effects on hindgut fermentation, digestibility, microbiome balance, reduction of pathogen shedding, and priming the immune system, among others [1,2,9,10,11,12,13,14,15,16] potentially mediated via multiple bioactive metabolites produced as a result of the fermentation process, residual yeast cells, and yeast cell wall components working singularly and/or in conjunction with each other [7]. Despite many examples of SCFP providing benefits to animal health and performance, for example in dogs [1,2,11], there is a lack of published research about the effects of SCFP on cats.

As in other species, the gastrointestinal tract of cats plays a crucial role in overall health, involving a complex interplay between the gut microbiota, immune system, and various physiological processes [17]. Recently, López Martí et al. [18] reviewed the current understanding of the impact of biotic solutions, including prebiotics, probiotics, and symbiotics, on feline gastrointestinal health. One aspect which was clearly missing was investigations into the impact of postbiotics. Simultaneously, given the unique physiological and dietary characteristics of cats, investigating the potential benefits of SCFP in this species is essential for advancing our understanding of its applicability as a nutritional intervention. Moreover, it is crucial to investigate whether the inclusion of SCFP would impact the palatability of diets for cats. This is particularly important because, contrary to dogs, in which the use of live yeast or derivatives seems to increase palatability [2,19,20], results in cats are not uniform, with no studies found specifically involving SCFP. Finally, to build our understanding of potentially considering SCFP as a postbiotic for cats, we need to understand the complex interaction between SCFP, bioactive compounds within it, and the feline host.

This study aims to fill a part of this gap by examining the effects of two inclusion levels of SCFP (Diamond V, Cedar Rapids, IA, USA) on diet palatability, fecal characteristics, apparent total tract macronutrient digestibility (ATTD), fecal fermentative end-products, fecal microbiota, and immune responses in adult felines over a period of 6 weeks.

## 2. Materials and Methods

### 2.1. Animals

Sixty-three Domestic Short-hair cats from a research colony, including 38 females and 25 males with body condition scores of 5.3 ± 0.6 [21], mean age of 6.3 ± 2.8 years, and mean body weight (BW) of 4.4 ± 0.1 kg (ranging 2.64–6.35 kg), were selected for this study. Inclusion criteria were no clinical signs and a non-remarkable physical exam at the time of the study. Exclusion criteria included signs of illness, inappetence, and uncooperative behavior with study procedures. Animals were individually housed in cages (0.64 m wide × 1.19 m long × 1.22 m high) in accordance with the Animal Welfare Act at the Summit Ridge Farms (Susquehanna, PA, USA). The light cycle was 12-h-light/12-h-dark, and every attempt was made to keep temperature ranges within targeted conditions (from 50 °F to 80 °F). Fresh tap water was available ad libitum via stainless-steel bowls.

### 2.2. Study Design and Diets

Prior to the study, all cats were fed the control kibble diet (CD) without SCFP (Table 1) during a 21-day acclimation period (day −21 to day 0). The amount of control diet offered during the acclimation period was calculated based on the metabolizable energy of the diet and energy requirements [100 × (BW kg)^0.67^] [22] of each individual animal’s initial body weight. After the first week, the amount of diet offered was adjusted weekly, if necessary, to maintain each animal’s body weight during the study.

After the acclimation period (day 1), the animals were randomly divided based on sex, body weight, body condition score, and age into three groups of 21 cats each to receive one of three experimental diets (continuing on CD, or transitioning to T150 or T300) for 42 days. The staff conducting the study were blinded to treatment. All three diets (Table 1) were dry extruded diets formulated to meet the nutritional requirements of healthy adult cats [23]. Diet T150 was CD with the addition of 1.0% of SCFP to achieve an approximate intake of 150 mg/kg body weight SCFP. Diet T300 was CD with the addition of 2.0% of SCFP to achieve an approximate intake of 300 mg/kg body weight SCFP. SCFP was incorporated into experimental diets prior to the extrusion process. SCFP is a dried product produced via *S. cerevisiae* fermentation that contains fermentation metabolites and some residual yeast cells. Nutrient analysis of the SCFP revealed that it contained 99% dry matter (DM), 72% ash, 8.6% CP, 0.7% crude fat, and 0.6% crude fiber.

Diets were offered once daily for a minimum of a four-hour period at approximately the same time each day of study. Remaining food was weighed at the end of the daily feeding period to determine daily food consumption.

Control diet: chicken mechanically deboned (inclusion 13.7%), chicken meal low ash (13.5%), brown rice (13.4%), oat groats (12.9%), pearled barley (12.9%), pea protein (12.7%), chicken fat (7.2%), menhaden fish meal (4.3%), powdered cellulose (2.2%), powder digest (1.8%), calcium carbonate (1.4%), choline chloride (0.8%), dicalcium phosphate (0.7%), potassium chloride (0.7%), mineral-vitamin premix (0.7%), taurine (0.4%), methionine (0.4%), potassium sulfate (0.2%), and mixed tocopherols (0.1%). All diets had the same ingredient composition, except calcium carbonate, brown rice, and dicalcium phosphate were adjusted and replaced by SCFP (TruMune®, Diamond V Mills™, Cedar Rapids, IA, USA). Profiles of amino acids, fatty acids, vitamins and minerals did not differ between the experimental diets.

### 2.3. Blood Sample Collection and Tests

Blood samples were collected on day 0 and day 40 for measurements of serum biochemistry (total protein, albumin, globulin, phosphorus, glucose, calcium, aspartate aminotransferase, alanine aminotransferase, alkaline phosphatase, γ-glutamyl transpeptidase, total bilirubin, blood urea nitrogen, creatinine, magnesium, sodium, potassium, chloride, cholesterol, triglycerides, creatine phosphokinase) and complete blood count (Antech, Fountain Valley, CA, USA).

The blood sample collected on day 40 was also utilized for analysis of oxidative stress markers [oxygen radical absorbance capacity (ORAC), malonaldehyde (MDA), superoxide dismutase (SOD), and thiobarbituric acid reactive substances (TBARS, which include MDA and other related compounds)] as well as Toll-like receptors (TLR) responsiveness.

Feline whole blood was processed, and peripheral blood monocytes (PBMCs) were isolated. Feline whole blood (K2-EDTA) tubes were placed on a rocker for 10 min. The whole blood was diluted 1:2 with 1X PBS and layered on 5 mL Ficoll-Paque. The blood was centrifuged for 30 min at 300× *g* at 4 °C with brake removed to isolate the PBMC. The PBMC layer was collected and washed with 10X volume of 1X PBS. An aliquot of the PBMCs was removed for counting with a hematology analyzer and the remainder underwent centrifugation at 300× *g* for 10 min. PBMC concentration was calculated, and the washed PBMC pellet was resuspended at 1 × 10^6^ cells/mL in complete growth medium (CGM). A total of 100 mL of the stock solution was seeded in the appropriate wells of a 96-well plate resulting in 1 × 10^5^ cells per well. Cells were incubated at 37 °C with 5% CO_2_ for one hour.

After 1 h of PBMC plating, 100 µL CGM was added to the media control and 100 µL of each test item was added to the appropriate wells. The effect of the test compound(s) on cell viability was determined by Alamar Blue assay: after 20 h of incubation, 22 µL of Alamar Blue was added and incubated for 4 h, then viability was assessed by plate reader. To evaluate TLR responsiveness, PBMCs were plated at 100,000/well and treated with four TLR agonists, which induced an inflammatory response. Cells were incubated for 24 h to observe any effects on TNF-α production. A custom TNF-α 1-plex was used to test the cell supernatants, collected 24 h after the test items were applied to the isolated feline PBMCs.

These analyses were conducted at the MLM Medical Laboratories (Oakdale, MN, USA). All test items were performed according to the manufacturer’s guidelines. Kits and reagents were selected for their broad use and validation in oxidative stress and immune function assays. Regarding the selection of TLR assays, TLR2, TLR3, TLR4, and TLR7/8 recognize key bacterial and viral components important for gut immune modulation, and were therefore relevant to SCFP and feline intestinal health. ORAC assay (ORAC/Antioxidant; category number STA-345) was supplied by Cell Biolabs (San Diego, CA, USA); MDA (General MDA ELISA kit; MBS2700234), MyBiosource (San Diego, CA, USA); SOD (SOD Activity Assay; 706002), Cayman Chemical (Ann Arbor, MI, USA); TBARS (colorimetric/fluorometric assay, 10009055), Cayman Chemical; TLR2 (zymosan, Tlrl-zyn), Invivogen (San Diego, CA, USA); TLR3 (Polyinosinic-polycytidylic acid sodium salt; P1530-25MG), Sigma Aldrich (St. Louis, MO, USA); TLR4 (lipopolysaccharides from *Escherichia coli* O55:B5; L5418-2ML), Sigma Aldrich; TLR7/8 (Imidazoquinoline compound—Resiquimond—TLR7/8 agonist; Vac-r848), Invivogen; and TNF-α (Multiplex 1-plex Feline Cytokine Mag; SPR2080), MilliporeSigma (Burlington, MA, USA).

### 2.4. Fecal Sample Collection and Fecal Characteristics

Naturally passed fecal samples were collected around day 0 (day −2 to day 0), day 21 (day 19 to day 21), and day 42 (day 40 to day 42). A three-day window was used to obtain one fresh fecal sample per cat, within 15 min of defecation. During the fecal collection days, litter pans were cleaned or changed after each observation and collection. Feces were collected, weighed, scored, pH measured, and aliquoted for conducting the other analyses. Fecal consistency was scored immediately upon collection, according to a five-point scale, with 1.0 = watery diarrhea, 1.5 = diarrhea, 2.0 = moist, no form, 2.5 = moist, poorly form, 3.0 = moist, formed, 3.5 = well formed, sticky, 4.0 = well formed, 4.5 = hard, dry, 5.0 = hard, dry, crumbly. The same trained personnel performed fecal scoring using a standard operating procedure, in order to reduce inter-observer variability. pH was measured immediately using a pH meter (Denver Instrument, Bohemia, NY, USA) equipped with an electrode (Beckman Instruments Inc., Fullerton, CA, USA).

For fecal IgA analysis, 1.5 g of fresh feces were placed in a cryotube that was immediately placed on dry ice, stored in a −80 °C until analyses by ELISA method (Immunology Consultants Laboratory, Inc., Portland, OR, USA) at the Animal Sciences Laboratory (University of Illinois Urbana-Champaign, Urbana, IL, USA). The fecal fermentative end-products measurements were carried out by gas chromatography at the Animal Sciences Laboratory (University of Illinois Urbana-Champaign, Urbana, IL, USA). Fecal concentrations of ammonia, short-chain fatty acids (SCFA; acetate, propionate, and butyrate), and branched-chain fatty acids (BCFA; valerate, isovalerate, and isobutyrate) were determined after preparing 5 g of each sample with equal amounts of 2N HCl:feces (e.g., 5 mL acid: 5 g feces) stored in a −20 °C freezer, as described previously [24]. To analyze fecal phenols and indoles, a minimum of 6 g of feces were stored in a −20 °C freezer [25] and fecal ammonia concentrations were determined according to the method of Chaney and Marbach [26].

### 2.5. Digestibility Test

The digestibility test for apparent total tract digestibility (ATTD) was conducted on day 34–39 with a total collection period of 120 consecutive hours, with the objective of collecting the feces for weight and analysis. Digestibility methods and analyses were performed according to Association of Official Analytical Chemists (AOAC) [27] and the Nutritional Guidelines for Complete and Complementary Pet Food for Cats and Dogs (FEDIAF) [23] approved analytical methodology for the following: moisture, crude fat, crude protein, fiber, ash, and gross energy. The ATTD coefficient of nutrients and energy was calculated using the following equation: digestibility (%) = [nutrient intake (g/day) − fecal output (g/day)]/nutrient intake (g/day) × 100%.

### 2.6. Shotgun Metagenomic Sequencing

267 frozen feline fecal samples preserved in 1 mL DNA/RNA shield were frozen and shipped to Cargill’s Minneapolis R&D Center (Plymouth, MN, USA) for shotgun metagenomic sequencing. Samples were thawed overnight at 4 °C, and genomic DNA extraction was performed using the ZymoBIOMICS 96 MagBead DNA kit (Zymo Research Corporation, Irvine, CA, USA) on the King Fisher Apex System (Thermo Fisher Scientific, Waltham, MA, USA). Due to the presence of PCR inhibitors in feline fecal samples, DNA was further purified using the ZR-96 Genomic DNA Clean and Concentrator-5 kit (Zymo Research Corporation, Irvine, CA, USA).

Nanopore sequencing libraries were constructed with the SQK-RPB004 Rapid PCR Barcoding kit (ONT, Oxford, UK). Samples that failed library preparation were cleaned again using the AMPure Beads (Beckman Coulter, Brea, CA, USA) following the manufacturer’s protocol, and library preparation was repeated for an additional two times. Seven samples failed troubleshooting, and were excluded. Despite using two different DNA extraction kits, these seven samples continued to exhibit inhibition during the library preparation step probably due to the presence of PCR inhibitors such as complex polysaccharides or phenolics, which are known to persist in feline fecal samples and inhibit enzymatic reactions. Shotgun metagenomic sequencing was performed using R9.4.1 FLO-MIN 106 flow cells on the GridION platform (ONT, Oxford, UK), multiplexing 10 samples in each flow cell. Blanks were included during library preparation for quality control, and each sequencing run (n = 25) lasted 70 h.

MinKNOW ONT software (v 5.2.8) with Guppy basecaller (v 6.2.11) was used for sequencing using the high accuracy base calling setting, followed by de-multiplexing, adapter trimming, and quality control using default settings.

#### 2.6.1. Metagenomics Taxonomic Assignation

Fastq files obtained from the MinKNOW ONT workflow were used for microbial taxonomic classification. First, host DNA was removed by mapping fastq files to the *Felis catus* genome assembly (Felis_catus_9.0) using Minimap2 [28], followed by the removal of reads matching the host genome (1.7 ± 5.7%) using Samtools v1.17 [29]. The remaining reads were assumed to be from microbial DNA and used for taxonomic assignation (364,856 reads ± 220,899).

Taxonomic assignation was performed using Kraken2 [30] with a custom database containing high quality genomes from the RefSeq database [31] and published metagenome-assembled genomes [31] classified with the GTBD taxonomy [32]. Estimation of abundance at the species, genus, family, and phylum level was performed with Bracken [33]. Using default settings, 87% (±4.5) of the sequences were able to be classified using the database.

#### 2.6.2. Functional Potential

To have a better understanding of the microbiome functional potential, gene orthologs, pathways annotated by KEGG [34], and Carbohydrate-active enzymes (CAZy) [35] were identified in the microbiome communities for each sample. First, genomes from microbial species identified with Kraken2 with relative abundance higher than 1 × 10^−4^, were annotated using PROKKA v1.1.1 [36], followed by additional assessment of gene function using EggNOG-mapper v2 [35]. After the annotation process was completed, an in-house python script was used to compile the KEGG Orthology genes (KOs), KEGG pathways, and CAZy for each genome, weighted based on the presence of each species abundance within the community, generating a table with the accumulated potential for all the microbes identified in each sample.

#### 2.6.3. Identification of Butyrate Producers

Microbial species identified in this dataset were queried for their potential to produce butyrate by exploring genome annotations for enzymes required for butyrate production through the acetyl-CoA pathway, glutarate pathway, and 4-aminobutyrate pathway (Table 2). This comprehensive approach yields a more complete identification of butyrate producers than simply targeting the terminal genes of the main butyrate-producing pathway [37,38].

Butyrate production likelihood was assigned to each species based on which genes in the pathway were detected. For the Acetyl-CoA route, detection of the last two enzymes in the pathway (1.3.1.86, 1.3.1.44) was required to give a genome a high likelihood of butyrate production via this route. For the glutarate and aminobutyrate route, all the genes in the pathway listed in Table 2 were required. If any genome representing the species showed the presence of these genes, the information was assigned to the entire species.

### 2.7. Palatability Tests

Standard 2-day tests were conducted using 20 healthy adult male and female cats (mean age = 8.4 ± 4.3 years; mean BW = 5.1 ± 1.3 kg) to compare all diet combinations (CD vs. T150, CD vs. T300 and T150 vs. T300). The test diets were presented to cats on an individual basis. Two stainless steel bowls, each containing approximately 100 g of the diet, were offered once daily for a duration of 4 h per day for 2 days. Bowl placement was reversed daily. If one diet was completely consumed prior to the end of the 4 h, both bowls were removed. Food consumption and first choice preference were recorded for each cat. The consumption of each diet was compared using a separate paired *t*-test within each day (20 cats by 2 diets) to assess significance in consumption difference for that day.

### 2.8. Data Processing and Statistical Analyses

All data, except for the palatability test and microbiota sequencing, were analyzed using R v4.2.2 (R Core team, Vienna, Austria 2022). Data management was conducted using the packages within “tidyverse” [39]; generalized linear models for statistical analyses were fit using the package “lme4” [40]; post-hoc contrasts conducted using the package “emmeans” [41]; and visualizations were created using the package “ggplot2” [42].

The treatment variable was coded in two different ways—an ordered variable, corresponding to the SCFP inclusion level in the experimental diets (0, 150 and 300 mg/kg), and as a binary variable comparing both SCFP inclusion levels (150 and 300 mg/kg) against the control diet (CD). Since both treatment and timepoint variables were ordered, orthogonal polynomial contrasts were implemented to assess the significance of trends with increasing dosage and trends over time in addition to pairwise comparisons between treatments and specific timepoints. Statistical significance was set as *p* < 0.05 and *p*-values were corrected for multiple hypothesis testing using the Benjamini-Hochberg false discovery-rate method.

Weekly observations of body weights were grouped into 2 periods (day 0 to day 21; day 22 to day 42), and the averages compared for each separately and overall (day 0 to day 42). Body weight was modelled using a linear mixed effects model, with treatment, period, and their interaction as fixed effects, an adjustment for cat sex, and cat ID as the random effect.

Comparisons of hematological parameters were made on the change in the parameter between the start and end of the trial. Cell counts and concentration parameters were compared between treatments using a mixed effects Poisson regression model, and concentration parameters with a mixed effects linear regression model. All models utilized treatment, timepoint, and their interaction as fixed effects, and cat ID as the random effect. The emmeans package [41] was used to estimate the change between day 0 and day 42 by computing the ratio (cells count at day 42/count at day 0) or the difference (concentration at day 42—concentration at day 0) for each treatment, and then these data were compared using pairwise and polynomial contrasts for treatment and dosage, respectively.

Immunological outcomes (blue cell viability, TNF-α agonists, MDA, SOD, TBARS and ORAC) were measured at a single timepoint and were log-transformed prior to fitting a linear mixed-effects model to assess treatment differences. The model included treatment (binary or ordered) as a fixed effect, with an adjustment for cat sex, and cat ID as the random effect.

Fecal indicators (phenols and indoles; pH; fecal score; fecal DM%; IgA; ammonia; SCFAs and BCFAs) were measured at multiple timepoints and were log-transformed prior to analysis if the observed distribution showed considerable deviations from normality. A linear mixed effects model was used to assess treatment differences, and included treatment, timepoint, and their interaction as fixed effects, with an adjustment for sex, and cat ID as the random effect. Dry matter, protein, fat, and energy digestibility measurements were not log-transformed prior to fitting a linear model with treatment and sex as predictors.

Regarding palatability, five analyses were employed to describe differences between each of the dietary comparisons, including an intake ratio of consumption using a Wilcox Signed Rank test and 2-way ANOVA to validate and test the assumption of a difference; overall and daily preference by first choice was determined using Chi-square (X^2^) probability to determine significance and average daily consumption using a paired *t*-test. Preference scoring by intake ratio to determine differences between preferences were assessed using a standard 2:1 consumption criterion, which is an accepted indicator of a clear preference. Acceptability was defined as feed intake divided by the total feed provided. As the outcome is a proportion, a mixed effects logistic regression was used to assess the association between probability of acceptance and treatment. The model included treatment, period, and their interaction as fixed effects, an adjustment for cat sex, and cat ID as the random effect. Differences between treatments were computed using the odds ratio (odds of acceptance in treated/odds of acceptance in control).

### 2.9. Metagenomic Outcomes

Before statistical analysis, rare taxa with relative abundance less than 1 × 10^−5^ were removed from the analysis, decreasing the dataset from 560 species to 346 species, and from 198 genera to 143 genera.

Alpha diversity metrics were calculated in R with the Phyloseq package [43] using species and genus count tables from Bracken as input and normalized first using rarefaction. Associations between treatment and alpha diversity were assessed for all observed taxa, as well as for the subset of potential butyrate producers described previously. Statistical comparisons for diversity metrics were conducted using linear mixed effects models with treatment, timepoint, and their interaction as fixed effects, and cat ID as a random effect.

Ordination for beta diversity analyses was performed using principal component analysis through the Phyloseq package on CLR-transformed taxon counts and Aitchison distance matrices. Permutational multivariate analysis of variance (PERMANOVA) using the computed distance matrices was performed with the adonis2 function in the R package vegan [44].

Differential abundance analysis was performed with the R package LinDA [45], modified to export all individual models for post-hoc contrasts. This analysis was performed on the subset of potential butyrate producing species, as well as the KEGG-KOs corresponding to the butyrate terminal reaction genes described in Table 2. The mixed model used the same formula as the alpha diversity analyses.

## 3. Results

### 3.1. Body Weight and Food Intake

The average daily food intake during the 42 days of the study was 66.2 (ranging 50–86), 65.1 (48–79), and 66.5 (52–85) g/day, for the CD, T150, and T300 diets, respectively, and was not different among the groups. Cat body weight (average: 4.38, 4.31 and 4.42 kg, respectively) was not different among treatment groups over the course of the study.

### 3.2. Blood Tests

All mean serum chemistry and blood cell count values were within the reference ranges. When comparing the difference between day 0 and day 42 between the groups, the only variable that showed any change was glycemia (Table 3). Blood glucose declined overall from 93.4 mg/dL (±2.03) at day 0 to 84.3 mg/dL (±1.90) at day 42, and declined significantly more in CD than T150 (*p* = 0.03), although the values were not significantly different between groups at either timepoint and were within the reference range. Although there was no difference in the common parameters of the complete blood count, when analyzing the ratio between the cell counts on day 42 and day 0, a linear and quadratic reduction in the day 42:day 0 ratio of absolute eosinophils and neutrophils was observed, as well as an increase in lymphocytes and monocytes, with no difference in the white blood cell count ratio day 42:day 0 regarding CD vs. T150 and T300 (Table 3).

Other immune and oxidative stress markers were measured (Table 3). Neither TLR responsiveness nor TBARS was altered by SCFP.

### 3.3. Fecal Characteristics

**Figure 1:**
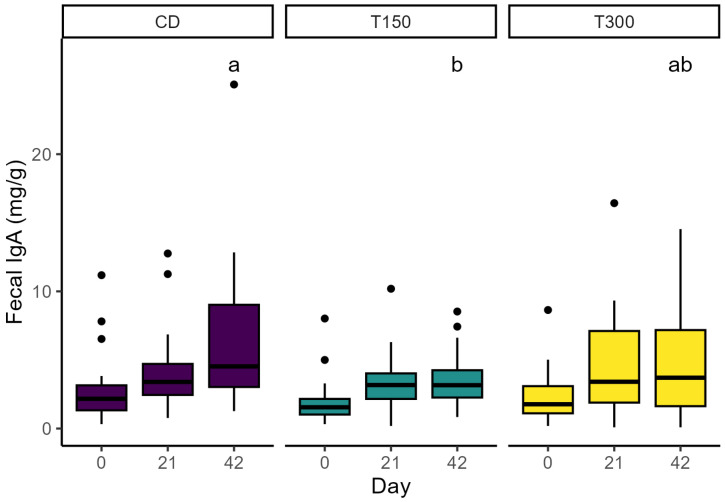
Fecal IgA concentration of cats (n = 63) before the dietary change (day 0), 21 days after, and 42 days after feeding the control (purple), T150 (green), and T300 (yellow) diets. Means within a day with different superscripts differ by *p* < 0.001.

Fecal pH was higher (*p* = 0.01) for SCFP-supplemented cats vs. CD and exhibited a positive linear dose response (*p* < 0.05) at day 21 and day 42. Fecal score was higher (firmer; *p* = 0.05) for the binary comparison of SCFP-supplemented cats vs. CD at day 21, although fecal dry matter content did not differ at this time (Table 4). Fecal butyrate decreased linearly with SCFP inclusion level (*p* < 0.001) and was lower (*p* < 0.05) for SCFP-supplemented vs. CD cats at day 21 and day 42. Total short-chain fatty acid (SCFA) was lower (*p* = 0.05) for SCFP-supplemented vs. CD cats at day 21. (Table 5). During total fecal collection (day 35–39), there was a linear decrease (*p* = 0.02) in the binary comparison of fecal moisture between CD vs. SCFP-supplemented cats (*p* = 0.01) (Table 6), but this was not accompanied by differences in the fecal score during this period (Table 4). Fecal IgA showed a quadratic treatment dose response on day 42 (*p* = 0.01), with cats fed T150 having the lowest fecal IgA values (Table 4, Figure 1). Details about the results of fecal characteristics and fermentation end products are available in Table 4 and Table 5.

### 3.4. Apparent Total Tract Macronutrient Digestibility

Supplementation of SCFP did not influence apparent total tract digestibility of DM crude protein, fat, or energy (Table 6). However, the feces of the animals receiving the SCFP had a slight linear decline in moisture, reflected in the comparison of the CD and SCFP groups (Table 6).

### 3.5. Palatability Test

In the two test days comparing CD versus T150, the food consumed by the 20 cats was 2687 g, with 988 g (36.8%) from CD and 1699 g (63.2%) from the T150 diet, resulting in a consumption ratio of T150:CD of 1.72:1. There was a significant consumption difference between the two diets (*p* = 0.01), even when accounting for the variance in consumption among the cats (F = 7.014, *p* = 0.02). Comparing the consumption of each diet per day, on day 1 the average daily consumption was higher from T150 than CD, while on the second day there was no difference (Table 7). The average Intake Ratios (IR) for each diet among the 20 individuals were 0.383 for CD and 0.617 for T150. Over both days, CD was chosen first 21 times and the T150 diet was chosen first 19 times. This result did not significantly differ from random choices (*p* = 0.75). Additionally, five cats consistently chose CD first every day, four cats consistently chose the T150 diet first every day, and the remaining eleven cats were undecided (*p* = 0.86).

In the comparison between CD and T300, the food consumed by the 20 cats totaled 2542 g, with 759 g (29.9%) from CD and 1783 g (70.1%) from the T300 diet, resulting in a consumption ratio of T300:CD of 2.35:1. There was a significant consumption difference between the two diets (*p* = 0.002), even when accounting for the variance in consumption among the cats (F = 14.539, *p* = 0.0012). The average daily consumption, compared on each day, was also higher with the supplemented diet than CD on day 1, with no difference on the second day (Table 7). The average IRs for each diet among the 20 individuals were 0.308 for CD and 0.692 for T300. Over both days, CD was chosen first 22 times and the T300 diet was chosen first 17 times. This result did not significantly differ from random choices (*p* = 0.42). Additionally, four cats consistently chose CD first every day, one cat consistently chose the T300 diet first every day, and the remaining fifteen cats were undecided (*p* = 0.05).

Lastly, in the comparison between the inclusion of SCFP, i.e., the T150 vs. T300 diet, the food consumed by the 20 cats totaled 2531 g, with 1484 g (58.6%) from T150 and 1047 g (41.4%) from T300, resulting in a consumption ratio of T150:T300 diet of 1.42:1. There was a significant consumption difference between the two diets (*p* = 0.01), even when accounting for the variance in consumption among the cats (F = 7.909, *p* = 0.01). The average IR for each diet among the 20 individuals were 0.596 for T150 and 0.404 for T300. In this comparison, the average daily consumption did not differ between the foods on day 1 (35.6 × 26.9 g/day; *p* = 0.27), but there was greater consumption of the T150 diet on day 2 than of the T300 (38.6 × 25.5 g/day; *p* = 0.03). Over both days, the T150 diet was chosen first 22 times and the T300 diet was chosen first 16 times. This result did not significantly differ from random choices (*p* = 0.32). Additionally, three cats consistently chose the T150 diet first every day, and no cats chose the T300 diet first every day, with the remaining seventeen cats being undecided (*p* = 0.005).

### 3.6. Alpha Diversity of the Fecal Microbiota

After filtering for the host genome, a total of 364,856 reads were obtained. The median sequencing count was 308,240 (±220,899; range 89,217–1,527,695).

There was no statistical difference in polynomial trend estimates of the association between Shannon diversity index and SCFP inclusion level stratified by sampling timepoint (Table S1), or between SCFP-supplemented and CD cats (Table S2). However, a polynomial trend estimate of the association between the Shannon diversity index and the sampling timepoint stratified by diet showed a significant linear decrease in diversity in CD compared to T150 and T300 (*p* = 0.04), where diversity was maintained at a similar level between day 0 and day 42 (Table 8).

When evaluating the Shannon diversity index on a subset of 30 butyrate producers, there was no significant trend observed on butyrate producers when stratified by sampling timepoint or by diet (Table S3).

**Figure 2:**
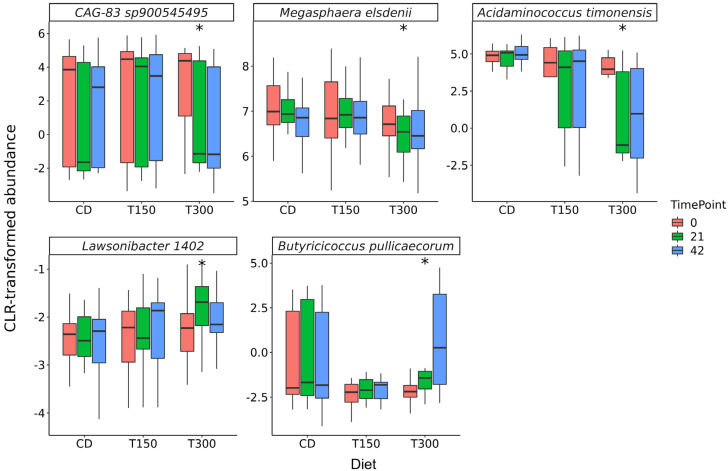
Differential abundance of butyrate producers in the fecal microbiota of cats, before the dietary change (day 0), 21 days after, and 42 days after feeding the control (CD), T150 and T300 diets. Within a microbial taxon, diets with significant linear trends between relative abundance and sampling timepoint stratified by diet are indicated with * (*p* < 0.05).

Differential abundance of butyrate producers showed that *Acidaminococcus timonensis* (*p* = 0.02), *CAG-83 sp900545495* (*p* = 0.02)*,* and *Megasphaera elsdenii* (*p* = 0.02) linearly decreased while *Butyricicoccus pullicaecorum* (*p* = 0.02) and *Lawsonibacter 1402* (*p* = 0.03) linearly increased over time for T300 cats (Figure 2, Table S4). No differences in abundance were detected for any other butyrate producing taxa in T300, nor between CD and T150 cats (Table S4).

### 3.7. Beta Diversity of the Fecal Microbiota

**Figure 3:**
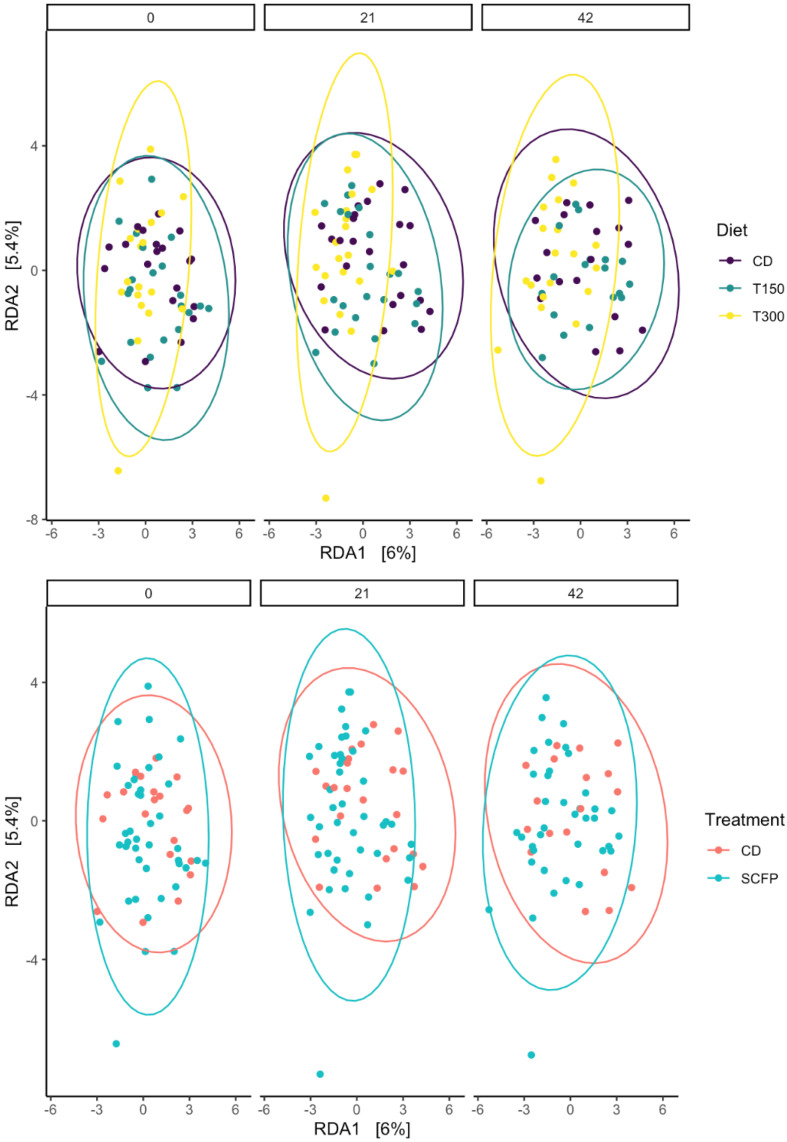
Beta diversity based on Centered Log-Ratio transformed taxon counts and Aitchison distance matrices, before the dietary change (day 0), 21 days after, and 42 days after (upper panel) using the control (purple), T150 (green), and T300 (yellow) diets, and (lower panel) feeding the control diet (pink) or diet enriched with *Saccharomyces cerevisae* fermentation product (SCFP; light blue).

Beta diversity based on Centered Log-Ratio-transformed taxon counts and Aitchison distance matrices is presented in Figure 3. Although the interaction between diet and timepoint was non-significant, there appears to be a significant association of both timepoint (*p* = 0.021) and diet (*p* = 0.001) with overall microbiome composition. This suggests that while the microbiomes of cats differed somewhat between diets, these differences were pre-existing at the start of the experiment. Furthermore, microbiome changes did occur over time, but the magnitude and direction of these changes did not differ significantly between different diets.

## 4. Discussion

Cats are gaining prominence in households and are considered the pets of the future due to the rising population of domestic felines, but are still underrepresented in terms of the amount of research and understanding when compared to dogs. Cat owners often have less access to nutritional and ingredient information, and this work seeks to improve upon that understanding. There is a demand for tools that improve companion animals’ lifespan and quality of life. Nutrition is one of the primary adjunctive tools in disease prevention as it is linked, among other aspects, to improved immune response, largely achieved through better intestinal health [46]. Postbiotics have emerged as a possibility for this [3], but there is little information for pets, and even less for cats as recently presented in a systematic review and meta-analysis [18]. While our previous research on postbiotics, specifically SCFP, has shown promise as a beneficial functional ingredient in dogs [2,11], little is known about the impact of SCFP on cats. In this study, we investigated the effects of increasing inclusion levels of SCFP in the diet of healthy cats on hematological, biochemical, and immunological variables, as well as digestibility, intestinal fermentation products, fecal microbiota, and palatability.

All cats remained healthy throughout the study. For certain biochemical parameters (glucose), there was a difference between the supplemented groups and the control group. Because this change was small and within reference ranges, and not accompanied by a change in the animals’ body condition status, it is likely not physiologically relevant. The potential causes of this change, including shifts in carbohydrate utilization by the microbiome or altered insulin response, were not investigated here, but could be addressed in future studies.

The maintenance of body condition score was ensured because the amount of food was controlled throughout the study. If the animals had been fed ad libitum, it was expected that the supplemented cats would exhibit higher food intake, given that in palatability tests, the diets enriched with SCFP were superior, particularly the T150. Palatability is complex and is affected by many different factors, including the preferences of individual animals; while these diets were generally preferred, this was not universally true for all individual cats. This is the first study to evaluate the palatability of SCFP for cats. It is noteworthy that the response appears to be species-specific, as the addition of SCFP to the diet did not alter palatability in dogs [2].

While the impact of SCFP on circulating immune cells and oxidative stress markers was assessed previously in dogs [13], little is known about the impact of SCFP on the immunity of cats. Additionally, in cats, changes in the circulatory immune cells and specific ratios such as the lymphocytes to monocytes ratio are increasingly being used as prognostic markers for certain disease states [47,48]. As an initial approach to assess the immune response, alterations in the leukocyte profile of the cats were observed. Comparing the differential leukocyte counts on day 0 and day 42, it was noted that there was a greater reduction in eosinophils and neutrophils, with an increase in monocytes and lymphocytes, while the total leukocyte count remained unchanged. A treatment that decreases eosinophils and neutrophils while increasing lymphocytes and monocytes may be modulating the immune response, potentially diminishing immediate inflammatory or allergic responses (eosinophils and neutrophils) and enhancing adaptive and long-term immune responses (lymphocytes and monocytes). This may be desirable in conditions such as allergies, autoimmune diseases, or certain viral infections, where modulation of the immune system could aid in disease management [49] and warrants future investigations. These observations are feline specific and differ from earlier studies looking into the impact of SCFP on immunity in dogs [13]. Other immune and oxidative status markers were also evaluated, showing no significant differences between treatments. A limitation of this and other studies on oxidative stress biomarkers is the fact that only serum markers were used, while urinary markers could also be included in future investigation [15].

As an additional immune parameter, in our study fecal IgA concentration was measured, which decreased after 42 days of treatment with SCFP supplementation. IgA is the most abundant class of antibodies found in the intestinal lumen, and it has long been recognized as a first line of defense in protecting the intestinal epithelium from enteric pathogens and toxins [50]. Physical and psychosocial stress can also impact IgA levels [51,52]. Accordingly, diet changes (from colony diet to study diet) and individual housing, amongst other factors, can impact fecal IgA levels. While the rate of increase of fecal IgA between the first 21 days of the study is similar across groups, after day 21, the rate of increase is lower in the SCFP supplemented groups. One possible hypothesis for this difference in fecal IgA levels is that SCFP supplementation may have mediated quicker adaptation to diet or other psychosocial stressors. Given that IgA production is stimulated by the immune response against antigens present in the intestine, it can also be hypothesized that a reduction in IgA may be related to a decreased physiological need for the secretion of this antibody, which can be interpreted as reduced intestinal inflammation [50,53]. We need further studies controlling for potential confounding factors, such as diet change and other factors contributing to psychosocial stress to better understand the connection between SCFA supplementation and fecal IgA in cats. Further investigations, including measurements for both fecal IgA as well as known markers of inflammation are required to shed light on the potential of SCFP-IgA mediated impact on intestinal inflammation status.

With the fecal collection, investigations were conducted on the impact of dietary SCFP on digestibility, which was not altered, as observed in a previous study with dogs [2]. Fecal quality underwent some transient modifications, namely an increased fecal score (firmer stools) in SCFP supplemented cats compared to CD at day 21, and reduced fecal moisture in the T300 group in days 35–39 during total fecal collection. However, these changes were small in magnitude, inconsistently observed by different measurement techniques, and did not persist through the end of the study, and as such may not represent a practical difference in stool quality. In a recent study with dogs [15], it was observed that the inclusion of SCFP in the diet minimized the increase in fecal moisture due to stress induction in the animals, which would be a protective effect against stress-induced diarrhea. Future studies in cats that include diet change or other stressors may add to the understanding of the effects of SCFP supplementation on fecal quality.

Regarding fecal characteristics, fecal pH values were significantly increased in supplemented cats. As in a previous study with dogs receiving a diet with hydrolyzed yeast, in which the same effect on pH was observed, it could be hypothesized that proteolytic fermentation with alkaline products prevailed over the production of acid products [54]. One potential confounding factor was the trend of pre-existing differences in fecal pH between groups at day 0 (quadratic *p* = 0.07). SCFP supplementation seemed to maintain a higher fecal pH over the course of the study compared to the control group. In all the groups, the pH was within the appropriate range for cats [55]. While learnings from other species would dictate a target of decreasing fecal pH as potentially beneficial the observed increase in pH with SCFP supplementation and its potential implications for feline physiology require further investigation.

The observed change in pH also corresponded to a non-significant increase in fecal ammonia concentration and lower fecal concentrations of SCFA and butyrate. While across-group comparisons revealed significantly lower fecal butyrate levels in SCFP-fed cats compared to controls at both day 21 and day 42, within-group comparisons revealed that butyrate levels were more or less stable within the SCFP-fed cats compared to the control group. This increase in the control group could hypothetically be attributed to a systemic need for a higher amount of butyrate in the control cats as they were still adapting to the change in the diet, study stress, or other confounding factors. In comparison, for the SCFP-fed cats, supplementation may have helped with the adaptation to the different stressors (diet change, study stress) by promoting a functionally able gut microbiome that met the systemic needs for butyrate. These within-group considerations might have contributed to the across-group observation of lower fecal butyrate in the SCFP-fed cats as compared to the control group. Other considerations such as changes in butyrate absorption, changes in transit time, amongst others, could also have impacted the measured levels of fecal butyrate. Unfortunately, these were not characterized in this study and warrant subsequent investigations.

In parallel with changes in butyrate, shifts in a functional subset of the microbiome were also observed in the T300 group. Fecal butyrate declined at day 21, concomitant with declines in several butyrate producers, then a significant increase in abundance of *Butyricicoccus pullicaecorum* at day 42 was accompanied by a return to baseline fecal butyrate levels. One hypothesis supporting this observation could be that of a re-organization of the butyrate producing microorganisms in cats on the T300 diet. Factors such as changes in butyrate production or absorption, amongst others, could also have contributed to the observed variation in fecal butyrate levels in the T300 group. Subsequent studies are warranted to investigate the relationship between microbes such as *Butyricicoccus pullicaecorum*, *Lawsonibacter* 1402, and fecal butyrate levels.

We also investigated the composition and dynamics of the overall microbiota in cats. Our findings revealed that the administration of SCFP contributed to the maintenance of fecal microbiome diversity, an indicator of gut health, in cats supplemented with SCFP. In contrast, cats that were not supplemented with SCFP exhibited a decrease in the Shannon diversity index over time. Notably, despite beginning the study with a lower baseline diversity, cats supplemented with SCFP maintained or improved their microbial diversity. Yeast-based postbiotics and inactivated yeast components are thought to produce a similar balancing effect on the microbiome via a diverse array of mechanisms, including antimicrobial activity against pathogens, yeast cell wall components serving as substrate for fermentation by SCFA-producing microbes, and inflammation reduction, highlighting the potential benefits of SCFP supplementation in preserving gut microbiota health [3,56,57,58].

We acknowledge several limitations in this study, including the selection of a singular breed, an absence of a defined challenge, and insufficient additional measurements, such as immunity and inflammation markers, amongst others. This limited our ability to obtain a complete picture of the impact of SCFP and the mechanistic functioning of SCFP supplementation. While this study provides a first viewpoint on the impact of SCFP in Domestic Short-hair cats, subsequent investigations across different breeds and different health and disease states could be beneficial to fully comprehend the potential postbiotic impact of SCFP in cats.

## 5. Conclusions

According to the results obtained in this study, SCFP may act as a functional ingredient in adult felines. SCFP had notable positive effects on diet palatability (especially 150 mg/kg BW), minimal impacts on stool quality, and no effect on total tract nutrient digestibility. Cats supplemented at 150 mg/kg BW had lower fecal IgA levels, and both levels of supplementation showed positive impacts on the circulatory leukocyte profile. SCFP helped preserve fecal microbiota diversity, but did maintain a higher fecal pH and lower fecal butyrate levels relative to control over a period of 6 weeks. Further investigations addressing the limitations of this study are required to fully understand the impact of SCFP on cats.

## Figures and Tables

**Table 1:** Nutritional profile of control (CD) and test diets used in the study.

Nutrient	CD	T150	T300
Moisture	7.6%	7.6%	7.6%
Crude protein (%DM)	33.0	33.8	34.7
Crude fat (%DM)	16.1	16.6	16.8
Crude fiber (%DM)	2.1	2.1	2.1
Ash (%DM)	7.5	7.7	7.9
Calcium (%DM)	1.6	1.6	1.7
Phosphorus (%DM)	1.0	1.1	1.1
Gross energy (kcal/kg DM)	4857	4887	4913

DM = dry matter.

**Table 2:** Enzyme ID and KEGG KO queried across all annotation files.

Butyrate Pathways	EC	Enzyme Name
Acetyl-CoA route	2.3.1.9	acetyl-CoA C-acetyltransferase
	2.8.3.5	3-oxoacid CoA-transferase
	1.1.1.35	3-hydroxyacyl-CoA dehydrogenase
	1.1.1.36	acetoacetyl-CoA reductase
	1.1.1.157	3-hydroxybutyryl-CoA dehydrogenase
	1.3.1.86	crotonyl-CoA reductase
	1.3.1.44	trans-2-enoyl-CoA reductase (NAD+)
Glutarate route	2.8.3.12	glutaconate CoA-transferase
	4.1.1.70	glutaconyl-CoA decarboxylase
Aminobutyrate route	1.1.1.6	4-hydroxybutyrate dehydrogenase
	4.2.1.120	4-hydroxybutanoyl-CoA dehydratase
	5.3.3.3	vinylacetyl-CoA Delta-isomerase
**Butyrate terminal reaction genes**	**KEGG KO**	
buk; butyrate kinase	K00929	
ptb; phosphate butyryltransferase	K00634	
atoD; acetate CoA/acetoacetate CoA-transferase alpha subunit	K01034	

**Table 3:** Blood characteristics and immune parameters of cats (n = 63) supplemented with *Sacharomyces cerevisae* fermentation product.

Parameter		Diets			SEM	*p*-Value		
		CD	T150	T300		Linear	Quadratic	CD vs. SCFP
Eosinophils count (ratio day 42/0)		0.88 ^a^	0.72 ^b^	0.71 ^bc^	0.01	**0.00**	**0.00**	**0.00**
Lymphocytes count (ratio day 42/0)		0.90 ^b^	0.91 ^ab^	0.95 ^c^	0.01	**0.00**	**0.00**	**0.00**
Monocytes (ratio day 42/0)		0.79 ^a^	0.99 ^b^	0.93 ^c^	0.02	**0.00**	**0.00**	**0.00**
Neutrophils (ratio day 42/0)		1.03 ^a^	0.98 ^b^	0.96 ^c^	0.01	**0.00**	**0.00**	**0.00**
White Blood Cell Count (ratio day 42/0)		0.98	0.95	0.94	0.09	0.80	0.90	0.77
Cholesterol (mg/dL; difference day 42-day 0)		2.29	5.48	12.59	4.12	0.09	0.70	0.19
Glucose (mg/dL; difference day 42-day 0)		−15.0	−4.95 ^d^	−7.46	3.16	0.09	0.11	**0.02**
TBARS ((log)uM)		1.66	1.85	1.75	0.06	0.33	0.08	0.09
ORAC (uM)		7118.24	7043.39	6400.52	593.91	0.39	0.69	0.58
SOD (U/mL)		0.51	0.51	0.54	0.03	0.42	0.69	0.62
MDA ((log)ng/mL)		8.11	8.04	7.92	0.11	0.20	0.86	0.30
ABCV [(log)Fluorescence Value]	Control	9.35	9.60	9.54	0.13	0.29	0.33	0.16
	TLR2	9.29	9.48	9.42		0.48	0.40	0.30
	TLR3	9.31	9.51	9.39		0.68	0.33	0.40
	TLR4	9.24	9.47	9.36		0.51	0.29	0.27
	TLR7/8	9.35	9.58	9.47		0.50	0.28	0.26
TNF-α [(log)pg/mL]	Control	1.63	1.93	1.71	0.20	0.77	0.29	0.43
	TLR2	3.36	3.72	3.63		0.35	0.36	0.20
	TLR3	3.10	3.09	3.28		0.53	0.68	0.73
	TLR4	2.93	3.07	3.27		0.23	0.92	0.32
	TLR7/8	5.21	5.24	5.41		0.47	0.78	0.63

ORAC = oxygen radical absorbance capacity; MDA = malonaldehyde; SOD = superoxide dismutase (SOD); TBARS = thiobarbituric acid reactive substances; ABCV = Alamar Blue Cell Viability. Ratio < 1 indicates a higher count at day 0, ratio = 1 indicates equivalent counts at both timepoints, and ratio > 1 indicates a higher count at day 42. ^abc^ Means with different superscripts differ *p* < 0.001. ^d^ CD vs. 150, *p* = 0.03. *p*-values in bold represent statistical significance (*p* < 0.05).

**Table 4:** Fecal parameters of cats (n = 63) supplemented with *Saccharomyces cerevisae* fermentation product.

Parameter	Timepoint (Day)	Diets			SEM	*p*-Value		
		CD	T150	T300		Linear	Quadratic	CD vs. SCFP
Fecal IgA (mg/g)	0 21 42	2.95 4.26 6.72 ^a^	2.07 3.43 3.54 ^b^	2.53 4.72 5.17 ^ab^	0.71	0.68 0.64 0.13	0.44 0.22 **0.01**	0.46 0.84 **0.01**
Fecal pH	0 21 42	6.10 6.01 5.84 ^c^	6.44 6.40 6.20 ^cd^	6.20 6.41 6.30 ^d^	0.13	0.61 **0.03** **0.02**	0.07 0.23 0.44	0.18 **0.01** **0.01**
Fecal Score ^w^	−7 0 21 42	3.11 2.63 2.51 2.65	2.93 2.89 2.83 2.52	3.14 2.78 2.79 2.59	0.13	0.89 0.39 0.11 0.75	0.20 0.23 0.25 0.52	0.60 0.18 **0.05** 0.54
Fecal Score ^w^ During Total Collection	35 36 37 38 39	2.57 2.76 2.71 2.69 2.64	2.69 2.81 2.82 2.77 2.86	2.68 2.85 2.77 2.86 2.95	0.15	0.59 0.66 0.77 0.41 0.15	0.71 0.98 0.63 1.00 0.71	0.51 0.69 0.62 0.47 0.15
Fecal DM, %	0 21 42	31.62 30.36 30.51	34.01 32.48 33.66	32.40 32.55 31.60	1.36	0.68 0.25 0.57	0.23 0.54 0.12	0.34 0.19 0.20
Fecal Ammonia, umol/g, DM Basis	0 21 42	176.92 159.24 239.20	214.53 222.13 202.36	185.09 185.44 221.03	24.75	0.81 0.44 0.60	0.26 0.09 0.35	0.44 0.13 0.35
Total Phenols, (log)ug/g, DM Basis	0 21 42	5.59 5.31 5.52	5.80 5.20 5.42	5.61 5.07 5.22	0.18	0.93 0.33 0.23	0.36 0.98 0.81	0.59 0.41 0.35
Total Indoles, (log)ug/g, DM Basis	0 21 42	5.10 5.16 5.04	5.15 4.83 4.86	4.99 5.18 5.24	0.22	0.70 0.94 0.48	0.69 0.19 0.26	0.85 0.60 0.94
Total Phenols/Indoles, (log)ug/g, DM Basis	0 21 42	6.09 5.85 5.89	6.18 5.73 5.86	6.10 5.87 5.97	0.16	0.97 0.96 0.94	0.62 0.47 0.54	0.78 0.75 0.71

^ab^ Means within a day with different superscripts differ *p* < 0.001. ^cd^ Means within a dose with different superscripts differ *p* < 0.05. ^w^ Fecal Score Subject Score Scale: 1.0 = watery diarrhea, 1.5 = diarrhea, 2.0 = moist, no form, 2.5 = moist, poorly form, 3.0 = moist, formed, 3.5 = well formed, sticky, 4.0 = well formed, 4.5 = hard, dry, 5.0 = hard, dry, crumbly. *p*-values in bold represent statistical significance (*p* < 0.05).

**Table 5:** Fecal short chain and branched chain fatty acids of cats (n = 63) supplemented with *Sacharomyces cerevisae* fermentation product.

Parameter	Timepoint (Day)	Diets			SEM	*p*-Value		
		CD	T150	T300		Linear	Quadratic	CD vs. SCFP
Acetate, (log)umol/g, DM Basis	0 21 42	5.10 5.51 5.52	5.35 5.37 5.42	5.41 ^a^ 5.39 5.44	0.08	**0.01** 0.27 0.47	0.33 0.38 0.53	**0.00** 0.17 0.34
Butyrate, (log)umol/g, DM Basis	0 21 42	4.10 4.41 4.43	4.15 4.22 4.28	4.14 3.93 ^b^ 4.09 ^c^	0.08	0.70 **0.00** **0.00**	0.76 0.56 0.80	0.63 **0.00** **0.01**
Propionate, (log)umol/g, DM Basis	0 21 42	4.70 4.75 4.71	4.56 4.60 4.64	4.72 4.72 4.74	0.08	0.92 0.79 0.79	0.12 0.15 0.39	0.49 0.35 0.84
Valerate, (log)umol/g, DM Basis	0 21 42	3.25 3.48 3.39	3.28 3.38 3.33	3.29 3.04 ^bd^ 3.11 ^e^	0.08	0.77 **0.00** **0.01**	0.93 0.22 0.40	0.77 **0.01** 0.09
Isobutyrate, (log)umol/g, DM Basis	0 21 42	1.85 1.71 1.78	1.91 1.77 1.78	1.86 1.80 1.80	0.08	0.89 0.45 0.88	0.59 0.91 0.94	0.70 0.47 0.93
Isovalerate, (log)umol/g, DM Basis	0 21 42	2.16 2.06 2.17	2.25 2.13 2.13	2.18 2.15 2.11	0.08	0.86 0.43 0.60	0.45 0.83 0.92	0.59 0.43 0.61
Total SCFA umol/g, DM Basis	0 21 42	401.01 519.45 527.96	444.56 445.32 449.48	458.61 435.97 449.48	33.26	0.22 0.08 0.10	0.72 0.43 0.67	0.21 **0.05** 0.10
Total BCFA umol/g, DM Basis	0 21 42	15.67 13.95 16.56	16.77 15.00 15.61	16.01 14.92 14.17	1.18	0.82 0.56 0.15	0.52 0.70 0.86	0.61 0.48 0.25

^a^ 0 vs. 300, *p* = 0.02; ^b^ 0 vs. 300, *p* < 0.00; ^c^ 0 vs. 300, *p* = 0.01; ^d^ 150 vs. 300, *p* = 0.01; ^e^ 150 vs. 300, *p* = 0.04. *p*-values in bold represent statistical significance (*p* < 0.05).

**Table 6:** Apparent total tract digestibility and fecal moisture during total collection phase of cats (n = 63) supplemented with *Sacharomyces cerevisae* fermentation product.

Parameter	SCFP (mg/kg BW)			SEM	*p*-Value		
	0	150	300		Linear	Quadratic	CD vs. SCFP
Fecal Moisture, %	71.41 ^a^	69.82 ^ab^	69.53 ^b^	0.56	**0.02**	0.35	**0.01**
Dry Matter ATTD, %	81.61	80.56	80.85	0.64	0.40	0.39	0.24
Protein ATTD, %	82.69	83.02	83.77	0.79	0.34	0.83	0.47
Fat ATTD, %	84.64	85.05	86.37	1.47	0.40	0.80	0.55
Energy ATTD, %	84.40	84.00	84.84	0.75	0.68	0.50	0.99

^ab^ Means with different superscripts differ *p* = 0.05. *p*-values in bold represent statistical significance (*p* < 0.05).

**Table 7:** Daily diet consumption and daily first choice of cats to compare all diet combinations.

Variable	CD × T150 Diets			CD × T300 Diets			T150 × T300 Diets		
	CD	T150	*p*-Value	CD	T300	*p*-Value	T150	T300	*p*-Value
Average Daily Consumption (g/cat/day) − day 1	20.5	44.2	**0.006** *	13.1	52.1	<0.001 *	35.6	26.9	0.272 *
Average Daily Consumption (g/cat/day) − day 2	29.0	40.8	0.200 *	24.9	37.1	0.211 *	38.6	25.5	**0.028** *
Daily First Choice (counts) − day 1	9	11	0.655 ^£^	8	12	0.371 ^£^	9	10	0.752 ^£^
Daily First Choice (counts) − day 2	12	8	0.371 ^£^	14	5	**0.043 ^£^**	13	6	0.114 ^£^

*p*-value obtained for: * separate paired *t*-test; ^£^ Chi-square test. *p*-values in bold represent statistical significance (*p* < 0.05).

**Table 8:** Shannon diversity index stratified by diet and timepoint, and polynomial trend estimates of the association between Shannon diversity index and sampling timepoint stratified by diet, of cats (n = 63) before the dietary change (day 0), 21 days after, and 42 days after feeding the control (CD) or supplemented diet with *Saccharomyces cerevisae* fermentation product.

Diets	Shannon Diversity Index [95% CI (Lower–Upper)] Timepoint (Day)			SEM	Polynomial Trend			
	Day 0	Day 21	Day 42		Contrast	Estimate	SE	*p*-Value
CD	3.34 (3.22–3.46)	3.28 (3.05–3.29)	3.20 (3.07–3.33)	0.06	Linear	−0.14	0.07	**0.04**
					Quadratic	−0.01	0.11	0.93
T150	3.17 (3.05–3.29)	3.23 (3.11–3.35)	3.14 (3.02–3.27)		Linear	−0.03	0.07	0.71
					Quadratic	−0.15	0.11	0.20
T300	3.24 (3.12–3.37)	3.27 (3.15–3.39)	3.29 (3.17–3.41)		Linear	0.05	0.07	0.48
					Quadratic	−0.01	0.12	0.90

CD = control diet; T150 = diet supplying *Saccharomyces cerevisae* fermentation product 150 mg/kg body weight; T300 = diet supplying *Saccharomyces cerevisae* fermentation product 300 mg/kg body weight. P-values in bold represent statistical significance (p<0.05).

## Data Availability

The raw data supporting the conclusions of this article will be made available by the authors on request.

## Supplementary Materials

The following supporting information can be downloaded at: https://www.mdpi.com/article/10.3390/ani15172551/s1, Table S1. Shannon diversity index stratified by timepoint and diet, and polynomial trend estimates of the association between Shannon diversity index and diet stratified by timepoint, of cats (n = 63) before the dietary change (day 0), 21 and 42 days after feeding the control (CD) or supplemented diet with *Saccharomyces cerevisae* fermentation product. Table S2. Shannon diversity index stratified by timepoint and treatment, and polynomial trend estimates of the association between Shannon diversity index and treatment stratified by timepoint, of cats (n = 63) before the dietary change (day 0), 21 and 42 days after feeding the control (CD) or supplemented diet with *Saccharomyces cerevisae* fermentation product (SCFP). Table S3. Shannon diversity index of butyrate producers stratified by diet and timepoint, and polynomial trend estimates of the association between Shannon diversity index of butyrate producers and sampling timepoint stratified by diet, of cats (n = 63) before the dietary change (day 0), 21 and 42 days after feeding the control (CD) or supplemented diet with *Saccharomyces cerevisae* fermentation product. Table S4. Polynomial trend estimates of the association between relative abundance of butyrate producers and sampling timepoint stratified by diet, of cats (n = 63) supplemented with *Saccharomyces cerevisae* fermentation product.
