# Supplementary material for: Effects of a Saccharomyces cerevisiae Fermentation Product on Diet Palatability and Feline Intestinal Health, Immunity, and Microbiome
Source: Animals (Basel). 2025 Aug 30;15(17):2551. doi: 10.3390/ani15172551 (PMC12427462; doi:10.3390/ani15172551)
Supplement: Supplementary file 1 [file animals-15-02551-s001.zip › animals-3665320-supplementary.pdf]

Supplementary Material

Table S1. Shannon diversity index stratified by timepoint and diet, and polynomial trend estimates of the association between Shannon diversity index and diet stratified by timepoint, of cats (n=63) before the dietary change (day 0), 21 and 42 days after feeding the control (CD) or supplemented diet with *Saccharomyces cerevisae* fermentation product.

**Timepoint (d)**	Shannon diversity index [95% CI (lower-upper)] **Diets**			**SEM**	Polynomial trend			
	CD	T150	T300		Contrast	Estimate	SE	P-value
0	3.34 (3.22-3.46)	3.17 (3.05-3.29)	3.24 (3.12-3.37)	0.06	Linear	-0.10	0.09	0.25
					Quadratic	0.25	0.15	0.10
21	3.28 (3.05-3.29)	3.23 (3.11-3.35)	3.27 (3.15-3.39)		Linear	0.00	0.08	0.97
					Quadratic	0.09	0.15	0.53
42	3.20 (3.07-3.33)	3.14 (3.02-3.27)	3.29 (3.17-3.41)		Linear	0.09	0.09	0.31
					Quadratic	0.21	0.16	0.19

CD = control diet; T150 = diet supplying *Saccharomyces cerevisae* fermentation product 150mg/kg body weight; T300 = diet supplying *Saccharomyces cerevisae* fermentation product 300mg/kg body weight.

Table S2. Shannon diversity index stratified by timepoint and treatment, and polynomial trend estimates of the association between Shannon diversity index and treatment stratified by timepoint, of cats (n=63) before the dietary change (day 0), 21 and 42 days after feeding the control (CD) or supplemented diet with *Saccharomyces cerevisae* fermentation product (SCFP).

**Timepoint (d)**	Shannon diversity index [95% CI (lower-upper)] **Treatment**			
	CD	SCFP	SE	P-value
0	3.34 (3.22-3.46)	3.20 (3.12-3.29)	0.08	0.07
21	3.28 (3.16-3.39)	3.25 (3.17-3.34)	0.07	0.73
42	3.20 (3.07-3.33)	3.22 (3.13-3.31)	0.08	0.81

CD = control diet; SCFP = diets supplying *Saccharomyces cerevisae* fermentation product at 150mg/kg body weight (T150) and at 300mg/kg body weight (T300).

Table S3. Shannon diversity index of butyrate producers stratified by diet and timepoint, and polynomial trend estimates of the association between Shannon diversity index of butyrate producers and sampling timepoint stratified by diet, of cats (n=63) before the dietary change (day 0), 21 and 42 days after feeding the control (CD) or supplemented diet with *Saccharomyces cerevisae* fermentation product.

**Diets**	Shannon diversity index [95% CI (lower-upper)] **Timepoint (d)**			**SEM**	Polynomial trend			
	Day 0	Day 21	Day 42		Contrast	Estimate	SE	P-value
CD	1.37	1.38	1.41	0.07	Linear	0.05	0.07	0.51
	(1.23-1.50)	(1.25-1.50)	(1.28-1.55)		Quadratic	0.02	0.12	0.85
T150	1.41	1.40	1.38		Linear	-0.03	0.07	0.67
	(1.29-1.54)	(1.27-1.53)	(1.25-1.52)		Quadratic	0.00	0.12	1.00
T300	1.44	1.49	1.56		Linear	0.13	0.07	0.08
	(1.30-1.57)	(1.36-1.62)	(1.43-1.70)		Quadratic	0.03	0.12	0.81

CD = control diet; T150 = diet supplying *Saccharomyces cerevisae* fermentation product 150mg/kg body weight; T300 = diet supplying *Saccharomyces cerevisae* fermentation product 300mg/kg body weight.

Table S4. Polynomial trend estimates of the association between relative abundance of butyrate producers and sampling timepoint stratified by diet, of cats (n=63) supplemented with *Saccharomyces cerevisae* fermentation product.

**Taxon**	**Diets**	Contrast	Estimate	SE	P-value	Adjusted P-value
Acidaminococcus massiliensis	CD	linear	0.71	0.44	0.11	0.63
		quadratic	0.98	0.73	0.18	0.65
	T150	linear	-0.62	0.44	0.16	0.65
		quadratic	0.24	0.73	0.74	0.81
	T300	linear	-0.37	0.44	0.4	0.76
		quadratic	0.08	0.74	0.92	0.94
Acidaminococcus sp000437815	CD	linear	-0.23	0.45	0.61	0.78
		quadratic	1.2	0.75	0.11	0.63
	T150	linear	-0.58	0.45	0.2	0.65
		quadratic	0.68	0.74	0.36	0.76
	T300	linear	-0.8	0.45	0.08	0.63
		quadratic	1.03	0.75	0.17	0.65
Acidaminococcus timonensis	CD	linear	-1.18	0.71	0.1	0.63
		quadratic	0.66	1.18	0.58	0.78
	T150	linear	-0.64	0.71	0.37	0.76
		quadratic	1.05	1.17	0.37	0.76

	T300	linear	-2.96	0.71	0	**0.01**
		quadratic	4.1	1.19	0	**0.02**
Agathobaculum desmolans	CD	linear	0.85	0.73	0.25	0.73
		quadratic	0.91	1.2	0.45	0.78
	T150	linear	-1.98	0.72	0.01	0.13
		quadratic	-0.69	1.2	0.57	0.78
	T300	linear	-1.05	0.73	0.15	0.65
		quadratic	0.69	1.22	0.57	0.78
Butyricicoccus pullicaecorum	CD	linear	-0.32	0.65	0.62	0.79
		quadratic	-0.73	1.07	0.5	0.78
	T150	linear	-0.32	0.64	0.62	0.78
		quadratic	-1.39	1.07	0.19	0.65
	T300	linear	2.4	0.65	0	**0.02**
		quadratic	0.89	1.08	0.41	0.76
CAG-81 sp000435795	CD	linear	-0.57	0.38	0.13	0.65
		quadratic	0.46	0.61	0.46	0.78
	T150	linear	-0.48	0.37	0.19	0.65
		quadratic	-0.69	0.62	0.27	0.73
	T300	linear	0.51	0.37	0.17	0.65
		quadratic	-0.43	0.63	0.49	0.78
CAG-83 sp900545495	CD	linear	-0.45	0.75	0.55	0.78
		quadratic	2.06	1.24	0.1	0.63
	T150	linear	-0.72	0.74	0.33	0.75
		quadratic	1.23	1.23	0.32	0.75
	T300	linear	-2.66	0.74	0	**0.02**
		quadratic	2.63	1.24	0.04	0.46
Clostridium_Q sp000435655	CD	linear	-0.18	0.16	0.26	0.73
		quadratic	0.29	0.26	0.26	0.73
	T150	linear	-0.11	0.16	0.47	0.78
		quadratic	-0.22	0.26	0.39	0.76

	T300	linear	0.18	0.16	0.26	0.73
		quadratic	0.28	0.26	0.28	0.73
Clostridium_Q sp003024715	CD	linear	-0.16	0.28	0.57	0.78
		quadratic	-0.18	0.46	0.7	0.80
	T150	linear	0.26	0.27	0.35	0.76
		quadratic	0.67	0.46	0.14	0.65
	T300	linear	-0.1	0.28	0.71	0.8
		quadratic	-0.25	0.46	0.59	0.78
Enterocloster aldenensis	CD	linear	-0.62	0.42	0.15	0.65
		quadratic	-1.05	0.7	0.13	0.65
	T150	linear	-0.07	0.42	0.86	0.91
		quadratic	0.3	0.7	0.67	0.8
	T300	linear	-0.38	0.42	0.36	0.76
		quadratic	-0.52	0.7	0.46	0.78
Evtepia gabavorous	CD	linear	0.22	0.33	0.5	0.78
		quadratic	0.78	0.53	0.14	0.65
	T150	linear	-0.13	0.32	0.69	0.8
		quadratic	0.3	0.53	0.57	0.78
	T300	linear	0.24	0.32	0.46	0.78
		quadratic	-0.59	0.54	0.28	0.73
Fusobacterium animalis	CD	linear	0.15	0.43	0.72	0.81
		quadratic	0.36	0.71	0.61	0.78
	T150	linear	-0.66	0.42	0.12	0.65
		quadratic	-0.1	0.71	0.88	0.93
	T300	linear	-0.46	0.43	0.28	0.73
		quadratic	0.63	0.71	0.38	0.76
Lawsonibacter 1402	CD	linear	-0.2	0.33	0.55	0.78
		quadratic	0.37	0.55	0.49	0.78
	T150	linear	-0.12	0.33	0.71	0.80
		quadratic	0.29	0.55	0.6	0.78

	T300	linear	1.09	0.33	0	**0.03**
		quadratic	0.22	0.55	0.69	0.80
Lawsonibacter 1775	CD	linear	0.91	0.64	0.16	0.65
		quadratic	-1.83	1.06	0.09	0.63
	T150	linear	-0.65	0.64	0.31	0.74
		quadratic	-0.6	1.06	0.57	0.78
	T300	linear	0.33	0.64	0.6	0.78
		quadratic	-0.35	1.07	0.74	0.81
Lawsonibacter 334	CD	linear	-1.33	1.02	0.2	0.65
		quadratic	0.21	1.67	0.9	0.94
	T150	linear	1.02	1	0.31	0.74
		quadratic	-0.33	1.67	0.85	0.90
	T300	linear	0.39	1.01	0.7	0.80
		quadratic	0.71	1.7	0.68	0.80
Lawsonibacter sp000177015	CD	linear	0.78	0.54	0.15	0.65
		quadratic	1.44	0.89	0.11	0.63
	T150	linear	0.5	0.53	0.35	0.76
		quadratic	0.18	0.89	0.84	0.90
	T300	linear	-0.52	0.53	0.33	0.75
		quadratic	1.56	0.9	0.08	0.63
Megasphaera elsdenii	CD	linear	-0.18	0.27	0.52	0.78
		quadratic	0.28	0.45	0.54	0.78
	T150	linear	-0.4	0.27	0.15	0.65
		quadratic	0.2	0.45	0.66	0.80
	T300	linear	-0.95	0.27	0	**0.02**
		quadratic	0.57	0.46	0.21	0.68
Megasphaera sp000417505	CD	linear	0.27	0.68	0.69	0.80
		quadratic	0.13	1.13	0.91	0.94
	T150	linear	0.81	0.68	0.24	0.73
		quadratic	-0.44	1.12	0.69	0.80

	T300	linear	-1.29	0.68	0.06	0.60
		quadratic	0.94	1.14	0.41	0.76
Megasphaera_B hexanoica	CD	linear	-0.22	0.4	0.57	0.78
		quadratic	-1.64	0.65	0.01	0.21
	T150	linear	-0.73	0.39	0.06	0.60
		quadratic	0.36	0.65	0.58	0.78
	T300	linear	0.17	0.39	0.67	0.80
		quadratic	0.01	0.66	0.99	0.99
Oscillibacter 130	CD	linear	-0.04	0.77	0.96	0.97
		quadratic	2.15	1.26	0.09	0.63
	T150	linear	-1.81	0.76	0.02	0.27
		quadratic	1.29	1.26	0.31	0.74
	T300	linear	0.28	0.76	0.71	0.8
		quadratic	1.63	1.28	0.21	0.67
Oscillibacter 218	CD	linear	-0.21	0.18	0.26	0.73
		quadratic	0.4	0.3	0.19	0.65
	T150	linear	-0.13	0.18	0.47	0.78
		quadratic	0.3	0.3	0.31	0.74
	T300	linear	-0.04	0.18	0.81	0.88
		quadratic	-0.17	0.3	0.57	0.78
Oscillibacter sp900548505	CD	linear	0.1	0.53	0.85	0.90
		quadratic	0.52	0.88	0.55	0.78
	T150	linear	0.43	0.52	0.41	0.76
		quadratic	2.64	0.87	0	0.06
	T300	linear	0.03	0.52	0.96	0.97
		quadratic	0.66	0.88	0.45	0.78
Oscillibacter welbionis	CD	linear	-0.2	0.3	0.51	0.78
		quadratic	0.37	0.49	0.45	0.78
	T150	linear	-0.12	0.29	0.69	0.80
		quadratic	0.29	0.49	0.56	0.78

	T300	linear	0.7	0.3	0.02	0.27
		quadratic	0.54	0.5	0.29	0.73
Schaedlerella 2052	CD	linear	-0.16	0.42	0.7	0.80
		quadratic	0.44	0.7	0.54	0.78
	T150	linear	-0.35	0.42	0.41	0.76
		quadratic	0.64	0.7	0.36	0.76
	T300	linear	0.84	0.42	0.05	0.52
		quadratic	0.05	0.71	0.94	0.96
UBA1191 sp900549125	CD	linear	-0.46	0.41	0.27	0.73
		quadratic	1.34	0.67	0.05	0.52
	T150	linear	-0.38	0.41	0.35	0.76
		quadratic	0.55	0.68	0.42	0.76
	T300	linear	-0.71	0.41	0.08	0.63
		quadratic	0.37	0.69	0.59	0.78

CD = control diet; T150 = diet supplying *Saccharomyces cerevisae* fermentation product 150mg/kg body weight; T300 = diet supplying *Saccharomyces cerevisae* fermentation product 300mg/kg body weight.
